# Unraveling the dynamics of loneliness in the Baltic-Nordic region: a comparative analysis in the wake of COVID-19

**DOI:** 10.3389/fpubh.2024.1360285

**Published:** 2024-04-22

**Authors:** Ieva Reine, Madara Miķelsone, Signe Tomsone, Helgi Guðmundsson, Andrejs Ivanovs, Halldór S. Guðmundsson, Ilze Koroļeva

**Affiliations:** ^1^Statistics Unit, Riga Stradiņš University, Riga, Latvia; ^2^Department of Public Health and Caring Sciences, Uppsala University, Uppsala, Sweden; ^3^Faculty of Rehabilitation, Riga Stradiņš University, Riga, Latvia; ^4^Social Science Research Institute, University of Iceland, Reykjavik, Iceland; ^5^Faculty of Social Work, University of Iceland, Reykjavik, Iceland; ^6^Institute of Philosophy and Sociology, Latvia University, Riga, Latvia

**Keywords:** SHARE, loneliness, demographic, health, socio-economic status, Latvia, Iceland, older adult

## Abstract

**Introduction:**

The primary aim of this study is to thoroughly investigate the prevalence and determinants of loneliness among older adults in the Baltic-Nordic region. Utilizing high-quality data sources and employing a methodologically rigorous approach, the study endeavors to enhance our understanding of how loneliness manifests and varies across different cultural and socio-economic contexts within these regions. By identifying key factors influencing loneliness, including demographic, social, and economic variables, the research seeks to contribute significantly to the existing body of knowledge on loneliness and inform targeted public health strategies and interventions tailored to the unique needs of older adults in the Baltic and Nordic countries.

**Material and methods:**

This research, centered on older adults aged 67 and above within the Baltic-Nordic region, draws upon data from the Survey of Health, Ageing and Retirement in Europe (SHARE), specifically its eighth wave conducted between June and August 2020. The demographic analysis of this study covers a diverse sample of 5,313 participants from the Baltic and Nordic regions. Specifically, the sample includes 2,377 participants from Nordic countries, namely Sweden, Denmark, and Finland, and 2,936 from the Baltic countries of Estonia, Latvia, and Lithuania. The investigation extends to the financial well-being of households, involving an analysis of 3,925 individuals, with 1,748 from Nordic countries and 2,177 from Baltic countries. Although Iceland is categorized as a Nordic country, the analysis within this study is conducted separately due to the unavailability of SHARE data for this region. Instead, the HL20 study, focusing on the health and well-being of the older adult population in Iceland, contributes data for 1,033 respondents. This methodological distinction allows for a comprehensive understanding of regional differences, highlighting the importance of specialized approaches to examine the intricate dynamics of loneliness and well-being across the Baltic-Nordic region.

**Results:**

The study reveals significant regional variations in loneliness among older adults during the COVID-19 outbreak, with the Baltic countries (Estonia, Latvia, Lithuania) reporting a lower prevalence of loneliness compared to the Nordic countries (Sweden, Denmark, Finland). Iceland, while grouped with the Nordic countries, was analysed separately. Employment emerges as a key factor in reducing loneliness across all regions, suggesting the benefits of social interactions and structured routines. Gender and marital status significantly influence loneliness, with notable disparities in the Baltic region and smaller gaps in the Nordic countries, reflecting the impact of societal and cultural norms. Additionally, educational attainment and health status show varied associations with loneliness, highlighting the complex interplay of individual and societal factors in these regions.

## Introduction

At the heart of this study is the exploration of loneliness, a complex and subjective state where individuals perceive a discrepancy between desired and actual social connections. This encompasses not only the absence of meaningful personal bonds, often termed emotional loneliness, but also the perceived lack of a wider supportive social network, known as social loneliness (1–3). The Baltic and Nordic regions, with their unique socio-cultural landscapes, offer a distinct backdrop for investigating loneliness. These areas are characterized by strong social welfare systems and a cultural emphasis on independence, which play significant roles in shaping individuals’ experiences of loneliness. By delving into these regional peculiarities, this study aims to shed light on the multifaceted nature of loneliness and its varied implications for public health within these contexts.

Loneliness, a multifaceted public health issue, extends its impact beyond the realm of emotional well-being, significantly influencing physical and mental health, as well as the overall quality of life (1, 2, 4, 5). The dynamics of loneliness, along with its determinants, exhibit substantial variation across different regions and countries, necessitating a deeper understanding of its global and regional nuances (6).

This variation is particularly evident in the Baltic and Nordic countries, where a range of factors uniquely shapes the prevalence of loneliness among their population (4, 6–10). Factors such as robust social support networks, marital status, and household composition have been identified as protective against loneliness (6, 11). Nonetheless, loneliness emerges as a significant predictor of depressive symptoms and a key determinant in the erosion of quality of life, even when other factors are accounted for (12, 13). Moreover, its association with adverse health outcomes such as cognitive decline, depression, and increased mortality further underscores its severity (5).

In-depth studies in the Baltic and Nordic regions reveal the multifaceted nature of loneliness. Estonia and Latvia, for instance, experience elevated levels of loneliness attributed to financial hardships and disrupted family communications (4, 14). Latvia, in particular, has shown a linkage between increased loneliness and factors like heightened irritability and reduced familial contact (15–17).

Within the Nordic countries, the frequency of social interactions and living arrangements significantly influence the experience of loneliness. Lower social contact frequencies and solitary living arrangements are strongly associated with higher levels of loneliness (18). Each Nordic country also faces unique challenges; for example, concerns about neighborhood safety in Sweden and Denmark, and income-related worries in Sweden and Finland. Emotional support scarcity has been noted as a factor in Denmark, Finland, and Sweden (19).

As we delve deeper into the intricacies of loneliness in the Baltic and Nordic countries, a complex web of individual, societal, and cultural factors becomes evident. Research spanning 25 European countries indicates geographical distinctions in loneliness experiences, with Northern European countries typically reporting lower loneliness rates compared to their Eastern counterparts (3). When comparing these findings at a broader European level, the distinct nature of loneliness within the Baltic and Nordic regions becomes even more apparent. Nordic countries like Finland, Norway, Sweden, and Denmark generally report lower levels of loneliness compared to Eastern European countries such as Latvia, which exhibit the highest prevalence (9, 18).

A global meta-analysis spanning over 113 countries further corroborates these regional trends (20). Moreover, loneliness’s implications extend beyond emotional distress. In Sweden, for example, older individuals experiencing loneliness have shown a higher propensity to utilize outpatient healthcare services, highlighting loneliness as a significant factor in healthcare utilization and costs (21, 22).

The COVID-19 pandemic has brought additional attention to the vulnerability of ageing populations to loneliness, particularly in the Baltic states. In Latvia, increased anxiety and disrupted familial interactions have been identified as noteworthy predictors of loneliness among older adults (14, 23).

This article aims to explore and elucidate the complex dynamics of loneliness within the Baltic and Nordic countries. By examining various factors influencing loneliness, identifying at-risk populations, and proposing evidence-based interventions, we seek to contribute to the growing body of knowledge on this crucial public health issue.

A significant aspect of this study is to examine the results from Iceland, particularly in the context of loneliness, and compare them with findings from the Nordic countries (24). This comparison aims to determine if Iceland’s patterns align with those observed in the Nordic region or if they present distinct trends. Through this analysis, we hope to not only validate our methodological approach (16, 25) but also contribute to a deeper understanding of regional dynamics in these European countries (18). This effort reflects a broader aim to enhance the comparability of data across different national contexts, thereby enriching the scope and depth of regional studies.

### Primary objective and scope

The primary objective of our research is to undertake a thorough comparative analysis of loneliness across demographic, socio-economic, and health domains, with an emphasis on the impact of the COVID-19 pandemic in the Baltic-Nordic region. Given the scarcity of research in this area, our study aims to fill a significant gap in the understanding of loneliness among older adults in this region.

Building on the insights from our comparative study of loneliness in Latvia and Iceland (16, 25), we aim to extrapolate these findings to the wider Baltic-Nordic context. This extension is crucial in determining whether the patterns and determinants of loneliness identified in the initial Latvia-Iceland comparison are consistent and applicable across the broader region.

A key challenge in our study is the examination of loneliness factors within the individual Nordic countries, particularly due to the variance in databases used in each country. To overcome this, we are committed to developing and employing robust constructs that enable meaningful and reliable comparisons between the diverse datasets. These constructs are specifically designed to harmonize different data collection methods and metrics, ensuring the comparability and validity of our analysis.

By successfully addressing these challenges, we anticipate that our study will significantly enhance the understanding of the dynamics of loneliness in the Baltic-Nordic region, especially in light of the recent global health crisis. The findings are expected to inform the development of targeted interventions and policies, tailored to the unique needs and circumstances of the older adult population in this region. Ultimately, our research aims to contribute valuable insights into the prevalence and determinants of loneliness, facilitating informed and effective public health responses in the Baltic-Nordic context.

Our study is primarily guided by the question, “How do the patterns and determinants of loneliness among older adults during the COVID-19 pandemic compare across the Baltic and Nordic regions, and what implications do these findings have for public health policy and interventions in these areas?”

Building upon our preceding research (16, 25), we further explore whether the patterns of loneliness prevalence and its determinants, as observed in the Latvia-Iceland comparative study, are consistent across the broader Nordic-Baltic region and within individual Nordic countries. This inquiry is particularly significant in the context of the methodological challenges presented by the use of different databases. To address these research questions, our study will focus on harmonizing data from various sources, ensuring comparability of variables and constructs across the datasets. This harmonization is crucial for drawing valid conclusions from diverse data sources.

## Methodology

### Data sources and population

The data for this study were obtained from two sources utilizing secondary data: The Survey of Health, Ageing and Retirement in Europe (SHARE) for the Nordic countries and the Health and Life conditions of the population of Iceland aged 67 and older (HL20) study for Iceland.

Thus, this study draws upon robust data from two distinct sources to comprehensively assess the prevalence and determinants of loneliness among older adults in the Baltic-Nordic region. The research leveraged data from the eighth wave of the Survey of Health, Ageing, and Retirement in Europe (SHARE), conducted between June and August 2020. SHARE is internationally renowned for its rigorous data collection practices and its profound influence on elevating research standards in the realm of social sciences (26). SHARE’s significance transcends the boundaries of the European Union. Notably, it meticulously encompasses all EU member countries through stringent harmonization processes, ensuring the comparability and reliability of data across countries. Furthermore, SHARE contributes to a global network of related studies, extending its reach from the Americas to Eastern Asia, and thus enhancing its impact and relevance beyond the European context. The utilization of SHARE data not only ensured the comprehensive coverage of the study population but also upheld the highest standards in research and scientific data collection, aligning with SHARE’s reputation for excellence in these domains.

The study population included older adults aged 67 and above within the Baltic-Nordic region, drawing participants from the Nordic countries of Sweden, Denmark, and Finland, and the Baltic states of Estonia, Latvia, and Lithuania. The total sample comprised 5,313 individuals, with 2,377 from the Nordic countries and 2,936 from the Baltic states. The research also delved into the financial well-being of 3,925 participants, including 1,748 from the Nordic region and 2,177 from the Baltic states.

It is crucial to note the separate analysis of Iceland, a Nordic country, due to the lack of SHARE data availability. Instead, the HL20 study provided insights into the health and well-being of 1,033 older adult Icelandic individuals, enriching the study’s comparative perspective on regional differences in loneliness and well-being within the Baltic-Nordic area. Furthermore, to facilitate meaningful comparisons, this study narrowed its focus to respondents aged 67 and older, ensuring that the sample aligns with the age range studied in Iceland.

The HL20 study, known as “Health and Life conditions of the population of Iceland aged 67 and older,” was conducted over a three-month period from November 2020 to January 2021. The study targeted Icelandic citizens who had reached retirement age and employed a simple random sample approach, selecting 1,800 individuals from the national registry. Ultimately, 1,033 respondents completed the survey.

The overarching aim of the HL20 study was to explore various aspects of well-being among older individuals in Iceland. It delved into physical and mental health, social participation, and financial stability, offering comprehensive insights into the conditions of the older adult population in Iceland. This initiative was part of a broader endeavour to better understand the needs and challenges faced by ageing individuals in Iceland, contributing valuable data for public health planning and policy-making. The findings from the HL20 study have been instrumental in shaping our understanding of loneliness and its determinants, particularly within the Icelandic context.

In our analysis, we draw upon the insights provided by Rapeli et al. (24), offering a comparative perspective on the response of the Nordic countries, Iceland included, to the COVID-19 pandemic, with a particular focus on older adult care strategies. This will help us illustrate Iceland’s approach to maintaining social connections amidst pandemic restrictions. Guðmundsson (27) provides an analysis of the circumstances of senior citizens in Iceland in 2020, offering valuable data on how the pandemic and related containment measures affected the well-being and social isolation of the older population. By comparing these findings with those from the Baltic and Nordic regions, we can discern unique socio-cultural and economic factors influencing loneliness among the older population in Iceland.

In comparing older populations in Latvia and Iceland, we have employed a standardized approach to ensure data comparability. This involved individuals aged 67 and above, utilizing SHARE for Latvia and HL20 data for Iceland (16, 25). Key demographic variables—gender, age, marital status, education, employment, financial stability, and health—were harmonized. The categorization of responses was also restructured for consistency, as outlined in previous research (16). This methodological framework is not only insightful for Latvia and Iceland but is now being applied to other populations in the Nordic-Baltic region, expanding its scope and enriching the understanding of ageing across diverse cultural and socio-economic contexts.

In summary, by judiciously harmonizing data sources and adopting a consistent age criterion, this study has harnessed the power of SHARE alongside Icelandic data to facilitate a meaningful and insightful comparison of loneliness prevalence and associated factors among older adults in the Baltic-Nordic region and Iceland. This methodological approach ensures both the reliability of findings and the ability to draw insightful cross-country comparisons.

### Assessment of variables

Loneliness, as the outcome variable, was categorized based on the available measures in each participating country, reflecting the diversity in data collection approaches. For countries utilizing the SHARE (Survey of Health, Ageing and Retirement in Europe) data, loneliness was assessed with the question, “How often do you feel lonely?” This allowed for a nuanced understanding of the frequency and intensity of loneliness experiences among respondents. In contrast, the loneliness indicator for Iceland, which relied on data from a different source, was determined by a more direct query, “Would you say that you are lonely?” This dichotomy in measurement approaches highlights the need to carefully consider and adapt to the specific methodologies employed in each country, ensuring that the loneliness assessments are both accurate and contextually relevant.

Demographic variables including gender, age groups, marital status, education, employment, financial situation, and health and wellbeing were standardized for comparative purposes. Health-related variables encompassed self-reported measures of physical and mental health status and self-assessment of change over time.

Response options underwent meticulous review and graphical arrangement to ensure consistency across countries. This standardization facilitated effective categorization of loneliness as an outcome variable, adhering to available measures in each country, enabling a coherent comparison.

These methodologies were selected for their reliability and validity in capturing the multifaceted nature of loneliness, mental health, and physical health within the studied population, aiming to achieve a comprehensive understanding of the factors influencing loneliness and its association with mental and physical health.

### Statistical analysis

Statistical analyses were conducted using IBM SPSS Statistics v.27, Jamovi v. 2.3.18, and R v.4.3.0. We began with descriptive statistics to summarize the characteristics of the study population and the distribution of key variables. This provided an overview of the sample and highlighted preliminary patterns in the data.

For inferential analyses, we employed a combination of techniques to examine the associations between loneliness and the variables of interest. The Pearson Chi-square test was utilized to assess categorical variables, allowing us to explore relationships between socio-demographic factors and loneliness levels.

## Results

### Feelings of loneliness and demographic factors in the Baltic region, Nordic countries, and Iceland

**Figure 1:**
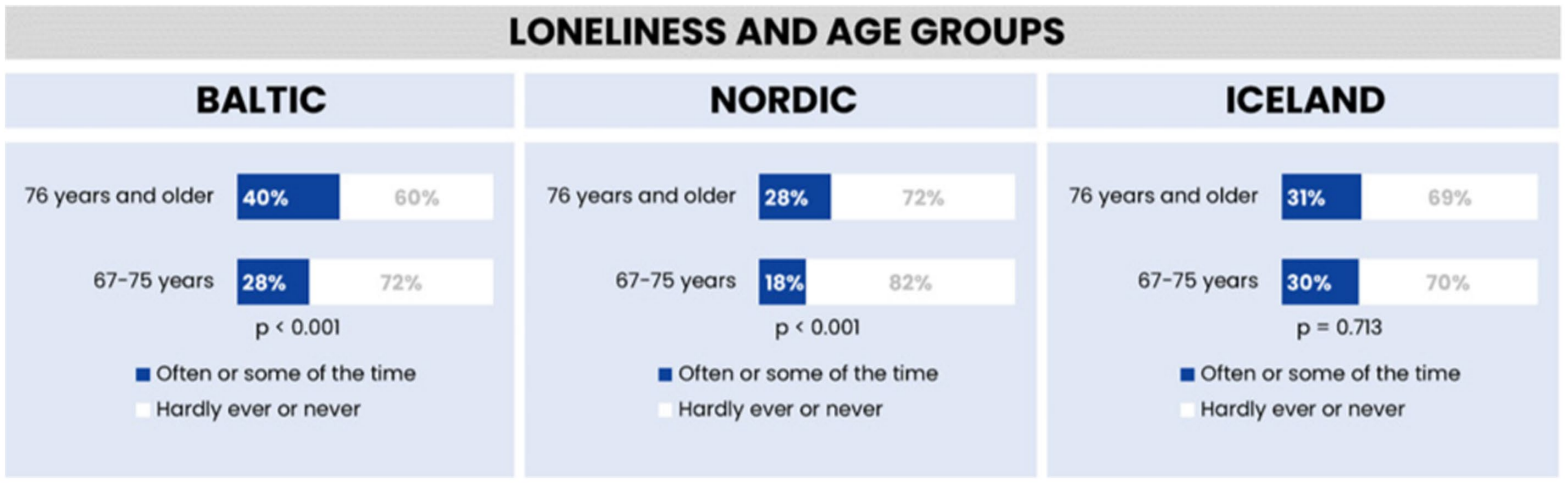
Feelings of loneliness across different age groups in the Baltic region, Nordic countries, and Iceland.

Figure 1 illustrates the distribution of loneliness among older adults in the Baltic region, Nordic countries, and Iceland, divided into two age brackets: 67–75 years and those 76 years and above. In the Baltic region, 40% of the older age group and 28% of the younger group report experiencing loneliness often or sometimes. The Nordic countries show lower prevalence, with 28% of the older group and 18% of the younger group reporting loneliness. In Iceland, the rates are similar across age groups, with about 30% reporting loneliness.

Statistical analysis confirms the significance of these findings for the Baltic and Nordic regions. However, the Icelandic data, with its higher *p*-value, indicates less certainty about the age-related differences in loneliness.

Both the Nordic and Baltic regions show a statistically significant difference in loneliness between the two age groups, with a greater proportion of individuals aged 76 and above reporting loneliness compared to the 67–75 age group. This trend is consistent in both regions, albeit with slightly lower percentages in the Nordic countries.

Conversely, Iceland’s data does not show a significant difference between age groups, with roughly 30% of individuals in both age brackets reporting loneliness. This suggests a more uniform experience of loneliness across ages in Iceland, unlike the Baltic and Nordic regions where age-related differences are more pronounced.

Therefore, while the Baltic and Nordic regions share similar patterns in how age influences loneliness, the actual percentages reveal that the loneliness experience in the Nordic countries is closer to that of Iceland, marked by a less pronounced age-related disparity.

**Figure 2:**
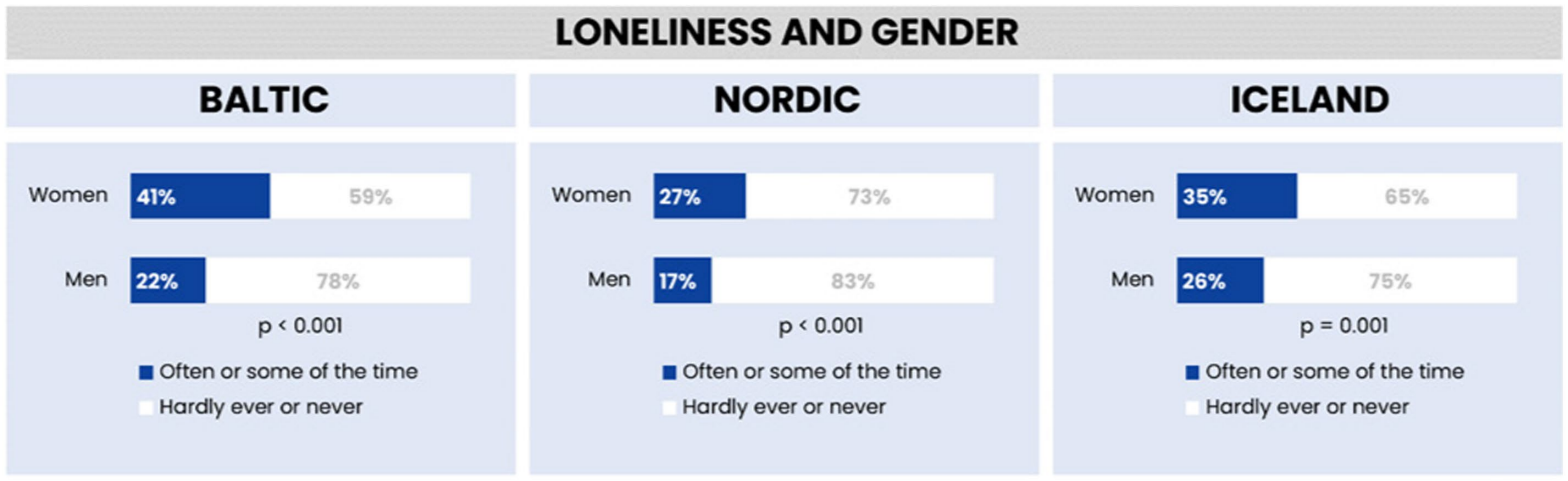
Feelings of loneliness by gender in the Baltic region, Nordic countries, and Iceland.

Figure 2 illustrates the gender-based differences in loneliness within the Baltic region, Nordic countries, and Iceland. The data indicate that women consistently report higher levels of loneliness compared to men across these regions. Specifically, in the Baltic region, 41% of women report often feeling lonely, in contrast to 22% of men. In the Nordic countries, the figures are 27% for women and 17% for men, while in Iceland, 35% of women and 26% of men report feelings of loneliness. These differences are statistically significant, highlighting a gender disparity in loneliness, with the smallest gap observed in Iceland.

Our statistical analysis across the Baltic region, Nordic countries, and Iceland revealed distinct patterns of loneliness influenced by gender and marital status. The analysis showed that, across all regions, women are more likely to report feelings of loneliness compared to men. The gender disparity in reported loneliness was most pronounced in the Baltic region and least pronounced in Iceland, with the Nordic countries showing intermediate levels.

Furthermore, the relationship between marital status and loneliness was examined, revealing significant variations across the three regions. In the Baltic region, there was a notable difference in reported loneliness between individuals living with a partner (19% reported feeling lonely) and those not living with a partner (49% reported feeling lonely). The Nordic countries exhibited a similar trend, though with lower overall percentages (15% for those living with partners and 38% for those not living with partners). In Iceland, while the percentage of those living with partners reporting loneliness (24%) was higher than in the Nordic countries, the pattern remained consistent, with higher loneliness reported among those not living with partners (44%).

**Figure 3:**
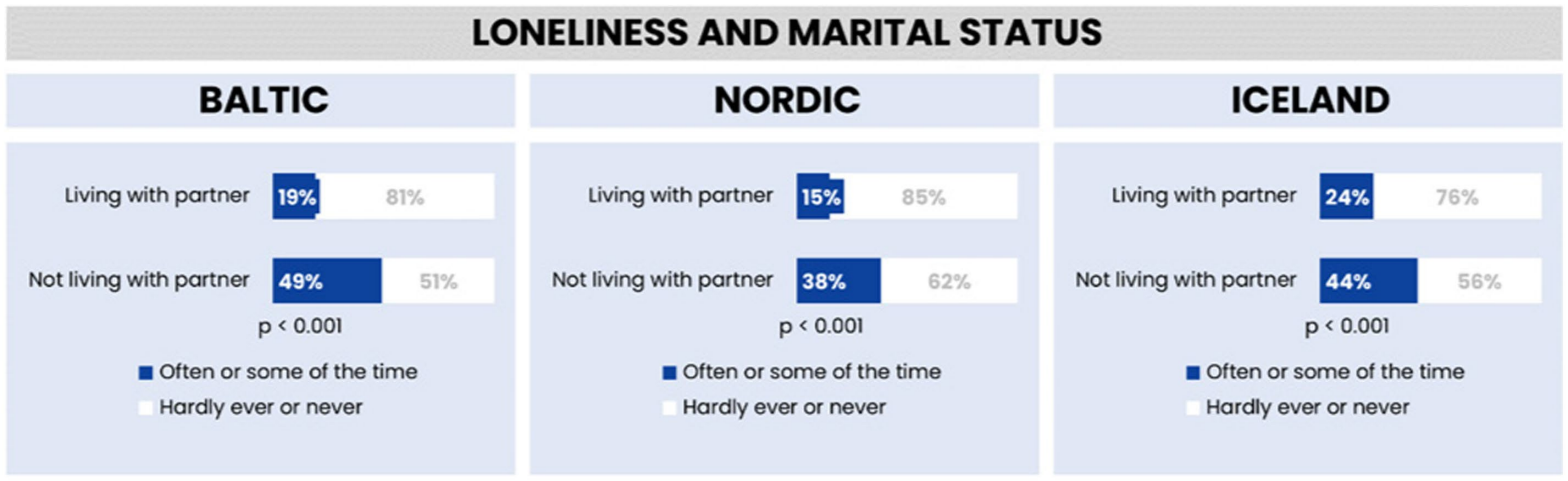
Feelings of loneliness by marital status in the Baltic region, Nordic countries, and Iceland.

The statistical significance of these findings was confirmed with a *p*-value of less than 0.001 across all regions, indicating a robust association between marital status and loneliness. This suggests that living with a partner may be associated with lower levels of reported loneliness, pointing to the potential protective role of cohabitation against loneliness (see Figure 3).

The analysis of loneliness in relation to marital status across the Baltic region, Nordic countries, and Iceland reveals distinct regional variations, highlighting the influence of different social and cultural contexts.

In the Baltic region, the disparity in loneliness between those living with and without a partner is particularly striking. Nearly half (49%) of individuals not living with a partner report feeling lonely often or some of the time, a rate significantly higher than that observed in the Nordic countries and Iceland. This high level of loneliness among single individuals could reflect cultural, social, or economic factors unique to the Baltic region. In contrast, those living with a partner in this region report the lowest rate of loneliness compared to their counterparts in the other regions, indicating the strong protective effect of cohabitation against feelings of loneliness.

The Nordic countries display the lowest overall loneliness rates. Here, only 15% of those living with a partner and 38% of those not living with a partner report feeling lonely. These lower rates across both groups suggest the presence of robust social support systems in these countries, which may help mitigate feelings of loneliness regardless of marital status. The Nordic region’s emphasis on social welfare, community support, and overall high quality of life could contribute to these lower rates of loneliness.

Iceland occupies a middle ground, with 24% of individuals living with a partner reporting loneliness, the highest rate for this group among the three regions. However, when considering individuals not living with a partner, Iceland’s rate of loneliness is lower than in the Baltic region yet higher than in the Nordic countries. This pattern might reflect Iceland’s unique social fabric, where tight-knit communities possibly play a significant role in influencing feelings of loneliness.

These findings collectively underscore the complex relationship between marital status and loneliness, which varies significantly across different geographic and cultural settings. The differences point to the need for region-specific strategies in addressing loneliness, taking into consideration the unique social dynamics and support systems present in each area.

### Feelings of loneliness and socio-economic factors in the Baltic region, Nordic countries, and Iceland

**Figure 4:**
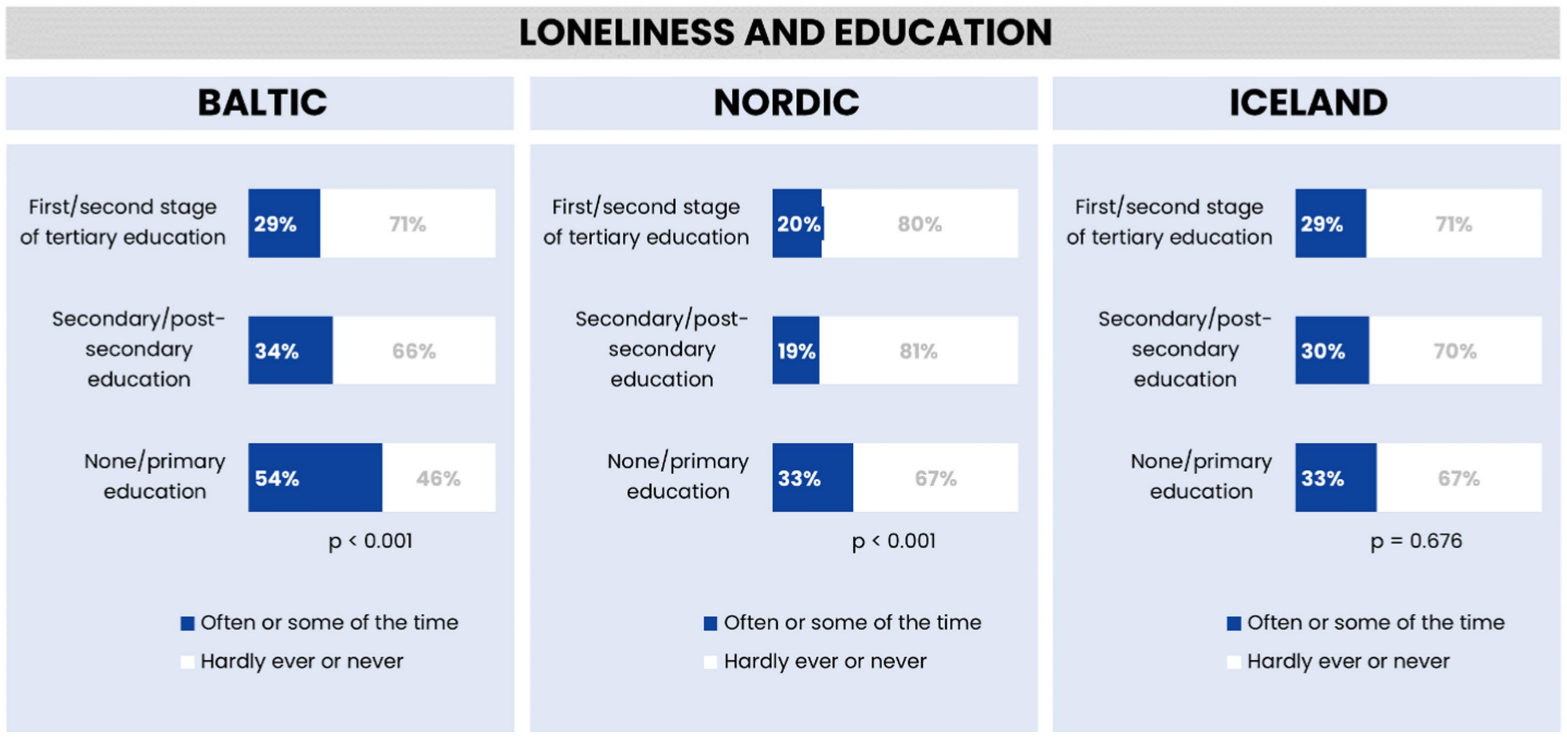
Feelings of loneliness by education in the Baltic region, Nordic countries, and Iceland.

Figure 4 shows an association between educational levels and loneliness in the Baltic region, Nordic countries, and Iceland. Higher levels of education are linked to lower reports of loneliness in the Baltic and Nordic regions. In the Baltic, those with no or primary education report the highest loneliness at 54%, followed by those with secondary education at 34%, and tertiary educated individuals at 29%. A similar trend is observed in the Nordic countries, with 33% of the least educated feeling lonely, 19% with secondary education, and 20% with tertiary education. In contrast, Iceland shows an equal percentage of loneliness (33%) among those with no or primary education and secondary education, with a slightly lower rate (29%) for those with tertiary education, but the differences are not statistically significant, suggesting education may not be a strong factor in loneliness there.

The Nordic countries have the lowest reported loneliness among the highly educated (20%) compared to the Baltic and Iceland (both at 29%). Statistically, education seems to affect loneliness in the Baltic and Nordic regions, but not significantly in Iceland. The Nordic region generally shows less loneliness across all education levels, hinting at stronger social support systems.

**Figure 5:**
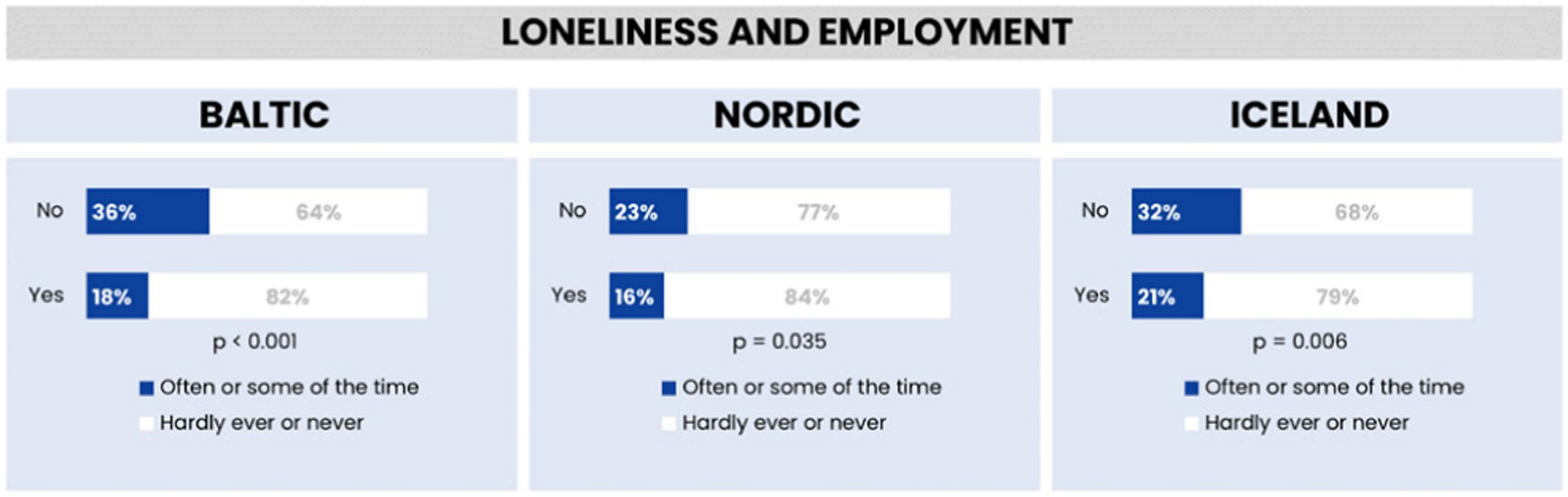
Feelings of loneliness by employment in the Baltic region, Nordic countries, and Iceland.

Figure 5 provides a summary of loneliness prevalence in the Baltic, Nordic, and Icelandic regions, illustrating a spectrum of loneliness experiences among the populations. A majority of respondents in each region report experiencing loneliness infrequently, with a smaller portion feeling lonely more regularly.

**Figure 6:**
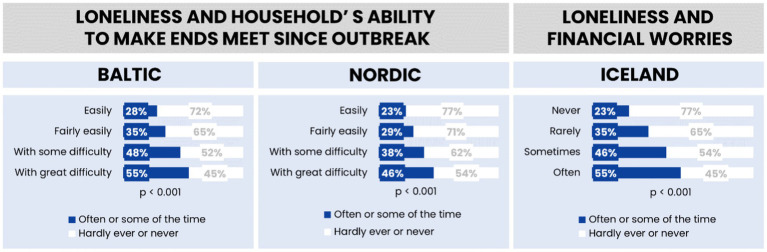
Feelings of loneliness by the household’s ability to make the ends meet/financial worries in the Baltic region, Nordic countries, and Iceland.

The analysis highlights regional differences in the occurrence of occasional and frequent loneliness, confirming the statistical significance of these findings with *p*-values less than 0.05. This indicates a clear variation in loneliness experiences across the regions, underscoring the nuanced nature of this emotional state within different cultural and geographic contexts. Detailed percentages and comparisons across the regions are depicted in Figure 6 providing a comprehensive visual representation of the data.

The data highlights the adverse effects of financial stress on feelings of isolation and social disconnection, underscoring the need for interventions aimed at improving financial security among vulnerable populations. These findings contribute to the existing body of research on the impact of economic factors on mental health and social relationships, reinforcing the importance of addressing financial stability as a key determinant of loneliness.

Figure 6 demonstrates a significant relationship between financial worries and loneliness in the Baltic and Nordic countries. The analysis indicates that individuals experiencing financial concerns report higher levels of loneliness compared to those without such worries. This trend is consistent across all countries studied, with statistical significance confirmed, suggesting that financial stability plays a crucial role in social well-being.

### Feelings of loneliness and physical health in the Baltic region, Nordic countries, and Iceland before the COVID-19 outbreak

**Figure 7:**
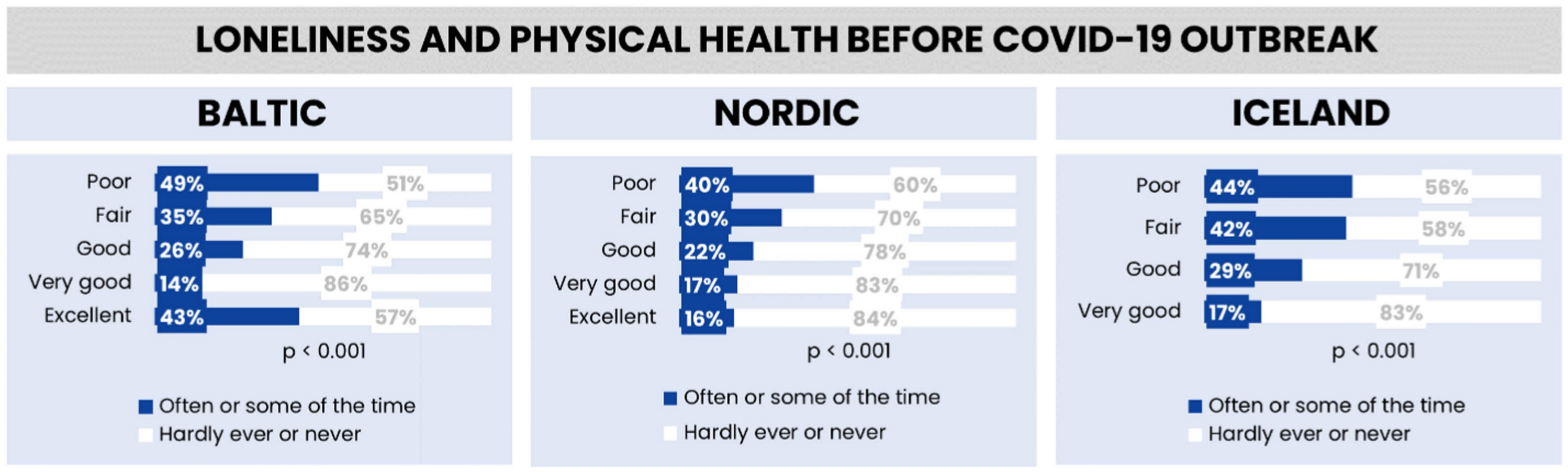
Feelings of loneliness by employment in the Baltic region, Nordic countries, and Iceland.

Figure 7 compares self-assessed physical health and the prevalence of loneliness in three different regions before the COVID-19 pandemic. In all three regions, individuals who rate their health as “Excellent” tend to feel lonely less often, with more than half reporting they hardly ever or never feel lonely. Conversely, a significant portion of those who rate their health as “Poor” tend to feel lonely more often. This trend is consistent across the Baltic, Nordic, and Iceland graphs, indicating a clear association between better self-perceived physical health and lower levels of loneliness.

The data is statistically significant for each region, strongly suggesting that the observed relationship between physical health and the frequency of loneliness is not due to random chance. This implies that in these regions, there is a notable association between how healthy people feel and how often they experience feelings of loneliness.

When comparing the Baltic, Nordic regions, and Iceland, the data reveals that the Baltic region reports the highest levels of loneliness among those with “Poor” and “Excellent” physical health. The Nordic region exhibits lower levels of loneliness across health categories compared to the Baltic region. Iceland shows the lowest percentage of loneliness in the “Excellent” health category but has levels similar to the Baltic region for those with “Poor” health. Overall, as the health status improves from “Poor” to “Excellent”, the frequency of loneliness decreases in all regions, with the Nordic region and Iceland showing a stronger negative correlation between good physical health and loneliness than the Baltic region.

### Feelings of loneliness and health in the Baltic region, Nordic countries, and Iceland during the COVID-19 outbreak

**Figure 8:**
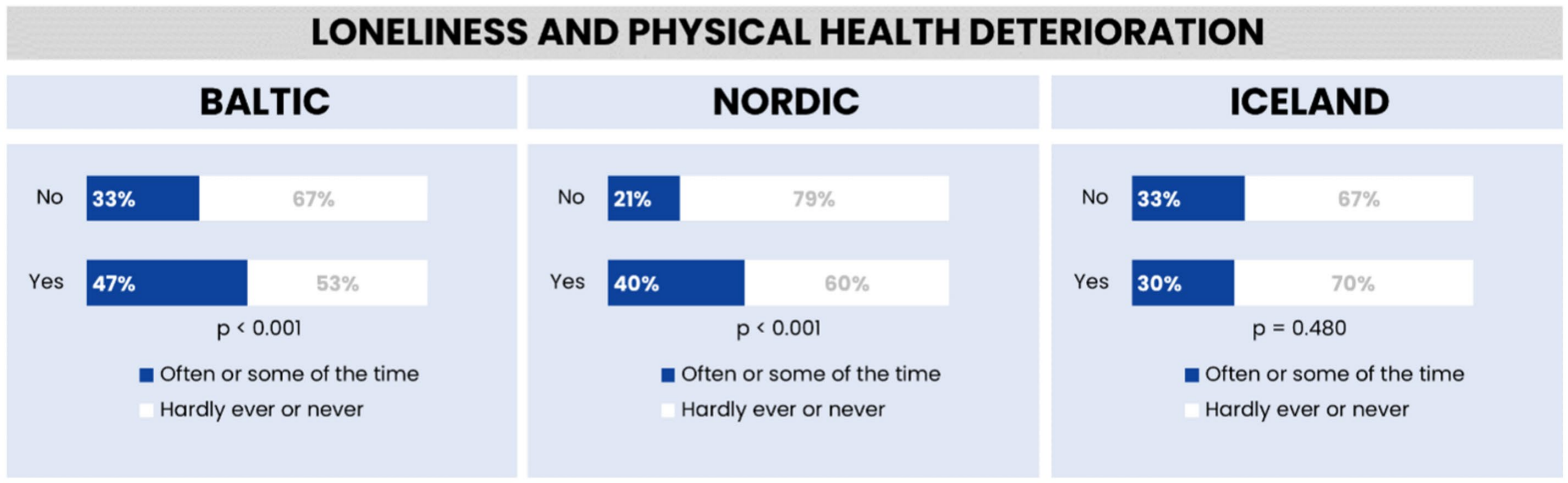
Feelings of loneliness and physical health deterioration in the Baltic region, Nordic countries, and Iceland.

Figure 8 presents an analysis of the relationship between physical health deterioration and loneliness across the Baltic region, Nordic countries, and Iceland. The data illustrates a notable trend where individuals experiencing health deterioration report higher levels of loneliness compared to those without such deterioration in the Baltic and Nordic regions. This association is statistically significant in these areas, indicating a strong link between health deterioration and increased feelings of loneliness. Conversely, in Iceland, the relationship between health status and loneliness does not show statistical significance, suggesting that other factors may be more influential in the experience of loneliness within this region.

**Figure 9:**
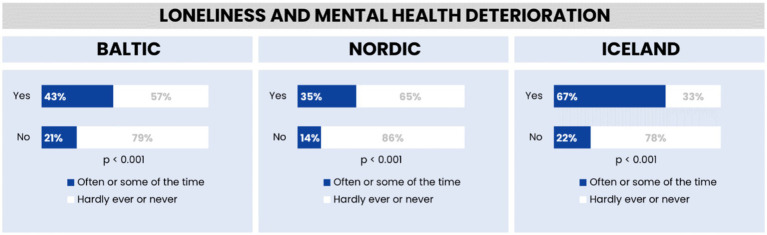
Feelings of loneliness and mental health deterioration in the Baltic region, Nordic countries, and Iceland.

The results in Figure 9 indicate that Iceland has the highest reported levels of loneliness, with 67% of individuals who reported a decline in mental health after the COVID-19 pandemic began (*n* = 185) felling lonely often or sometimes. This is in contrast to 43% in the Baltic region and 35% in the Nordic region. In terms of linking loneliness with mental health deterioration, 86% of respondents in the Nordic region acknowledge this correlation, compared to 78% in Iceland and 79% in the Baltic region. The association between loneliness and mental health deterioration is statistically significant across all regions.

## Discussion

This study presents a comparative analysis of loneliness in relation to employment across the Baltic, Nordic regions, uncovering regional variations that are statistically significant. Our methodological approach, integrating diverse data sets, including the distinct Icelandic data and the SHARE survey data, has set a new benchmark for future comparative studies.

In the Baltic region, fewer reports of loneliness may reflect cultural or economic factors fostering community and belonging, as supported by the strong statistical significance of our findings. The Nordic countries, known for their high quality of life and robust social welfare, report the lowest levels of loneliness, potentially due to social trust and community engagement (3, 10). Iceland, despite a higher proportion of loneliness, shows a majority still feeling seldom lonely, influenced possibly by its tight-knit communities (16).

Employment appears to play a beneficial role in reducing loneliness across all regions, associated with structured routines and social interactions (28). However, the cross-sectional nature of our data precludes definitive conclusions about causation between employment and loneliness, indicating a direction for future longitudinal studies (29).

Our findings also highlight the need for region-specific strategies to combat loneliness, particularly concerning employment. The differences in the prevalence and experience of loneliness in the Baltic, Nordic, and Icelandic regions underscore the complexity of this social phenomenon.

Gender disparities in loneliness are notable. In the Baltic region, a higher rate of loneliness among women compared to men suggests potential societal and cultural influences (8). The Nordic countries exhibit a smaller gender gap in loneliness, possibly due to more inclusive social policies (30). Iceland’s closer rates of loneliness between genders may be attributed to its communal ties and social norms (31).

Marital status also correlates with loneliness. The Baltic region shows a higher loneliness rate among individuals not living with a partner, possibly due to social and economic transitions (12, 18). The Nordic countries, with their strong welfare policies, report lower loneliness rates regardless of marital status (32). Iceland’s unique social structure reflects the importance of broader social networks beyond intimate relationships (16, 33). These differences highlight how social and cultural factors, along with individual relationships, can influence feelings of loneliness. The significantly higher rates of loneliness in the Baltic region for those not living with a partner could reflect cultural, economic, or policy differences that might affect social connectedness and support systems. The Nordic countries seem to be doing better in terms of combating loneliness, potentially due to their emphasis on social welfare and community programs. In Iceland, the relatively higher rate of loneliness among those living with a partner compared to the Nordic countries might suggest different societal norms or expectations in personal relationships. Overall, marital status appears to be a strong indicator of loneliness across all regions, but its impact varies by region.

Educational attainment shows varied associations with loneliness across regions. An inverse correlation between education and loneliness in the Baltic and Nordic regions suggests protective benefits of higher education levels (8). However, in Iceland, education level does not significantly predict loneliness, indicating other influential factors (16).

Health status and loneliness also share a significant relationship. While the Baltic and Nordic regions show an inverse relationship between perceived health and loneliness (4, 8), Iceland’s findings suggest the importance of community bonds (16, 34).

The multifaceted nature of loneliness, as revealed by regional studies, underscores the critical interplay between individual emotional states, personality traits, and broader socio-economic and familial contexts. In countries like Estonia and Latvia, elevated loneliness levels, attributed to financial hardships and disrupted family communications, highlight the significant impact of socio-economic factors (4, 14). Moreover, the association of increased loneliness with heightened irritability in Latvia points to the influential role of emotional and personality characteristics in the experience of loneliness.

This study’s findings on loneliness across different regions emphasize the complexity of this issue, influenced by individual, cultural, and societal factors. These insights are vital for developing tailored public health strategies to address loneliness and its mental health implications.

## Limitations of the study

This study has several limitations that should be acknowledged. Firstly, the integration of distinct Icelandic data with the SHARE survey data may have introduced inconsistencies due to different data collection methods and structures. The study’s cross-sectional design limits the ability to establish causal relationships between factors like employment and loneliness, highlighting the need for longitudinal studies to explore these relationships over time. The findings are specific to the Baltic, Nordic, and Icelandic regions and may not be applicable to other contexts because of cultural, economic, and social differences.

Additionally, the study relies on self-reported measures, which could lead to biases as responses may be influenced by personal perceptions and cultural norms. Focusing predominantly on individuals aged 67 and above excludes younger age groups, who may have different experiences of loneliness. Cultural norms and gender roles within the surveyed regions could impact the reporting of loneliness, potentially leading to skewed results.

The data for this study were obtained from two primary sources: The Survey of Health, Ageing and Retirement in Europe (SHARE) for the Nordic countries and the Health and Life conditions of the population of Iceland aged 67 and older (HL20) study for Iceland. The sample sizes differed between the Baltic and Nordic countries, with 2,936 individuals from Baltic countries and 2,377 from Nordic countries. Despite the higher population in Nordic countries, the sample size was smaller, which merits discussion.

The disparity in sample sizes can be attributed to methodological constraints and the specific focus of the studies used. SHARE, being a multi-national project, includes various countries with different sampling frames and response rates, leading to variations in sample sizes across countries. Additionally, the HL20 study focused solely on Iceland and employed a simple random sample of 1,800 individuals from the national registry, with 1,033 respondents completing the survey. These differences reflect the specific research objectives and resources available for each study.

The disparities in sample sizes between Baltic and Nordic countries, while potentially introducing selection bias, were addressed through methodological adjustments. Statistical techniques, including weighted analyses, were employed to mitigate the impact of sample size differences, ensuring proportional contributions from each country’s data to the overall analysis. Additionally, harmonization of key demographic variables facilitated meaningful cross-country comparisons.

These methodological considerations provide a solid foundation for the study’s cross-country comparisons. However, it’s essential to acknowledge the limitations associated with sample size disparities and recognize the need for future research to strive for more balanced samples across countries to enhance the validity and generalizability of findings.

Regarding the issue of selection bias, while the random sampling approach and relatively high response rate minimize the risk, it’s essential to acknowledge potential biases, particularly among individuals residing in care facilities for older adults. Future research should explore strategies to mitigate such biases and further enhance the robustness of findings.

## Bidirectionality in relationships

An important consideration in interpreting the findings of our study is the potential bidirectionality of relationships, particularly for self-reported variables such as self-reported health. Bidirectionality refers to the reciprocal influence between variables, where the relationship between two factors may operate in both directions over time.

For example, while our analysis identified a significant association between self-reported health and loneliness, it’s essential to recognize that this relationship may be bidirectional. On one hand, poor self-reported health may contribute to increased feelings of loneliness, as individuals facing health challenges may experience social isolation, reduced mobility, or limitations in participating in social activities.

Conversely, loneliness itself can also impact self-reported health outcomes. Studies have shown that chronic loneliness is associated with adverse health consequences, including increased risk of chronic diseases, impaired immune function, and accelerated ageing. Therefore, individuals experiencing loneliness may be more likely to report poorer health status, reflecting the psychosocial impact of loneliness on overall well-being.

While bidirectionality provides important insights into the complex interplay between variables, it also poses challenges for causal inference and interpretation of findings. In our study, the bidirectional relationship between self-reported health and loneliness highlights the need for caution in drawing causal conclusions from the observed associations.

Moreover, the paucity of details provided in the methods section regarding the assessment of self-reported variables limits our ability to fully explore bidirectional relationships. Future research should incorporate longitudinal designs and more comprehensive measures to assess the temporal sequence and directionality of relationships between variables. The statistical methods used to align different data sets have inherent limitations and might not fully address all variations in the data. The study might not have accounted for all relevant factors influencing loneliness, such as individual health conditions or local community dynamics. Variations in social welfare policies and community support structures across the studied regions, which might affect experiences of loneliness, are not extensively examined. Finally, the representativeness of the study’s sample size for capturing a broad range of experiences related to loneliness might be limited. These limitations are important for understanding the scope of the study and for guiding future research in this area.

## Conclusion

While this study primarily focuses on the Baltic and Nordic regions, it contributes significantly to the broader understanding of loneliness as a global public health issue. The research illuminates the intricate nature of loneliness and its profound impact on both individuals and communities. By integrating various data sources, the study captures the nuanced experiences of loneliness in distinct geographical contexts, offering valuable insights that pave the way for future research and the formulation of public health initiatives aimed at mitigating loneliness.

The heightened loneliness observed among ageing populations in the Baltic states, particularly exacerbated by the ongoing COVID-19 pandemic, underscores the urgent need for targeted interventions. Policymakers and social workers must grasp the complexities of these findings to develop effective strategies for alleviating loneliness. Emphasis should be placed on creating programs tailored to the specific needs and challenges of different demographics within each region.

Future research endeavors should explore longitudinal studies to understand the temporal dynamics and causal relationships of loneliness. Such studies would provide insights into how loneliness evolves in response to changes in socio-demographic, economic, and health-related factors. Comparative studies across various cultural contexts are also crucial for identifying both universal and culture-specific factors that influence loneliness. This knowledge can guide the development of culturally sensitive interventions. Moreover, there is an essential need to develop, implement, and rigorously evaluate interventions aimed at reducing loneliness, especially among vulnerable populations, to enhance social connectedness.

The implications of this research extend beyond academic inquiry, offering practical guidance for public health policy, clinical practice, and community engagement. Insights from the study can inform the development of targeted public health policies and programs that promote social inclusion and reduce loneliness by addressing the identified determinants. Healthcare providers are encouraged to integrate loneliness screening and interventions into routine care practices, thus enhancing patient care by addressing the psychosocial aspects associated with loneliness. Community organizations and social services agencies can leverage the study’s findings to create initiatives that strengthen community bonds and promote social interaction, addressing loneliness at the grassroots level.

In conclusion, by highlighting the importance of loneliness as a public health issue, this study lays the groundwork for future research and practical interventions designed to alleviate loneliness and its associated challenges. This work contributes to the well-being of individuals in the Baltic and Nordic regions, especially during challenging times like the current pandemic, and has implications for addressing loneliness on a global scale.

## Data availability statement

The raw data supporting the conclusions of this article will be made available by the authors, without undue reservation.

## Author contributions

IR: Conceptualization, Data curation, Formal analysis, Funding acquisition, Investigation, Methodology, Project administration, Resources, Supervision, Validation, Writing – original draft, Writing – review & editing. MM: Data curation, Formal analysis, Investigation, Software, Validation, Visualization, Writing – original draft, Writing – review & editing. ST: Conceptualization, Supervision, Validation, Writing – original draft, Writing – review & editing. HeG: Formal analysis, Investigation, Methodology, Software, Writing – original draft, Writing – review & editing. AI: Conceptualization, Methodology, Supervision, Writing – original draft, Writing – review & editing. HaG: Conceptualization, Investigation, Supervision, Validation, Writing – original draft, Writing – review & editing. IK: Formal analysis, Investigation, Methodology, Writing – original draft, Writing – review & editing.
